# Vaccination of calves with Bacille Calmette Guerin increased the frequency but did not affect aggregation or clustering of natural killer cells in draining lymph nodes

**DOI:** 10.1093/discim/kyaf017

**Published:** 2025-11-13

**Authors:** Jayne C Hope, Sarah Ho, Clara Zifko, Carly A Hamilton, Darren J Shaw

**Affiliations:** The Roslin Institute, University of Edinburgh, Edinburgh, UK; The Roslin Institute, University of Edinburgh, Edinburgh, UK; The Roslin Institute, University of Edinburgh, Edinburgh, UK; The Roslin Institute, University of Edinburgh, Edinburgh, UK; The Roslin Institute, University of Edinburgh, Edinburgh, UK; Royal (Dick) School of Veterinary Studies, Edinburgh, UK

**Keywords:** Bacille Calmette Guerin vaccine, natural killer cells, bovine tuberculosis

## Abstract

**Introduction:**

Natural killer (NK) cells are central to innate immune responses but they also influence adaptive immunity. Evidence suggests that NK cells are involved in protective immune responses induced by the Bacille Calmette Guerin (BCG) vaccine. In cattle, vaccination with BCG provides significant protection against infection with *Mycobacterium bovis*, the causative agent of bovine tuberculosis (bTB). Bovine NK cells were previously shown to traffic from BCG vaccination sites in afferent lymph, and to be activated reciprocally through interactions with dendritic cells (DC) to drive high-level interferon gamma secretion. To further define roles for bovine NK cells in the induction of BCG vaccine-mediated immunity, we examined alterations in their frequency, location, and aggregation in lymph nodes (LN) draining immunization sites.

**Materials and Methods:**

Calves were either not vaccinated, vaccinated with BCG once, or were re-vaccinated. The frequency and localization of NK cells in draining LN was examined by immunohistochemistry and immunofluorescence, and statistical analyses of imaging outputs were performed.

**Results:**

While increased numbers of NK cells were found in BCG-draining LN, there were no significant alterations in location, nor the clustering or aggregation of NK cells. Re-vaccination with BCG had little impact on NK cell numbers or location.

**Conclusion:**

BCG vaccination induced changes in NK cell frequency in bovine LN. Further studies of NK cell function and co-localization with subsets of DC and T cells will be important to define the roles of these cells in the induction of protective immunity in bTB.

## Graphical Abstract

**Graphical Abstract:**
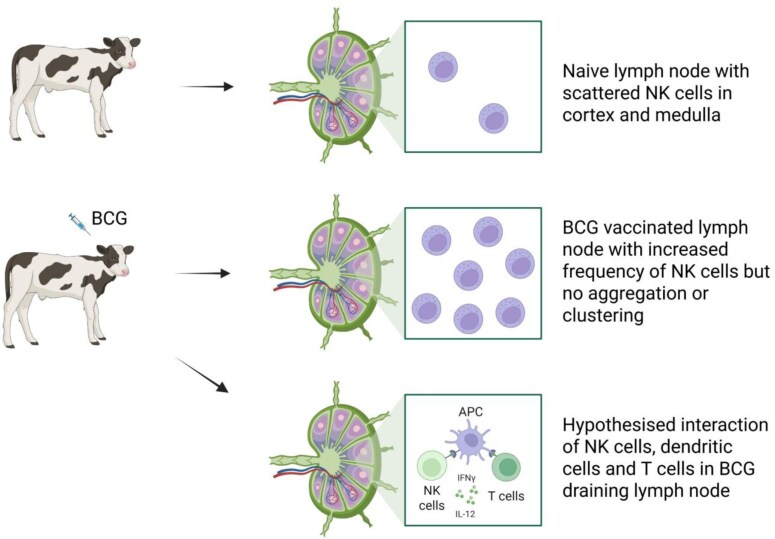
Impact of Bacille Calmette Guerin (BCG) vaccination on natural killer (NK) cell frequency in bovine lymph nodes (LN). In non-vaccinated calves, NK cells are scattered throughout the LN. Following vaccination with BCG, the frequency of NK cells increased, but their location within the LN was not altered. We hypothesize that increased numbers of NK cells increase the potential for cellular interactions that are central to adaptive (protective) immunity. Created in BioRender. Hope, J. (2025) https://BioRender.com/pg277n3

## Introduction

Natural killer (NK) cells are innate cells that respond rapidly during primary immune responses but are also known to influence adaptive immunity. In humans, mice and cattle NK cell subsets identified as CD56^bright^ or CD27^hi^ or CD2^−^ respectively, produce high levels of interferon gamma (IFNγ) and are present in greater numbers in lymph nodes (LN) compared with the peripheral blood [1–3]. In naïve laboratory mice, the frequency of NK cells is very low within LN (0.3–0.5%) [4–6]. However, in free-living wild mice, the proportion of NK cells in secondary lymphoid tissues is significantly higher, and these cells are more activated or primed [7].

In the steady state, murine LN NK cells are positioned predominantly in the medulla, as well as in the interfollicular regions neighbouring the subcapsular sinus, and at the boundary of the T cell zone and B-cell follicles [4, 8–11]. The resting NK cells exist in a motile state with a large proportion in contact with resident networks of dendritic cells (DCs) [4]. In bovine LN, NK cells are found in significant numbers within the parafollicular and medullary areas but are mostly absent in B-cell follicles. The relative numbers of NK cells in bovine LN [12] are similar to those reported for humans (1–7%) [2, 13]. The greater prevalence of NK cells within LN of cattle may reflect the higher environmental microbial burden to which cattle are exposed, resulting in a primed, resident population of NK cells [12].

The impact of inflammatory signals, vaccination, or infectious challenge on the recruitment of NK cells to the LN has been demonstrated in mice and humans [6, 14]. The recruited NK cells were shown to express IFNγ for T-cell priming and subsequent Th1 polarization [6, 15], with redistribution to more central regions of the parafollicular areas [8, 16] which likely influence T cell activation and interactions with DC. Reports of an intimate co-localization between NK cells, DC, and T cells in LN suggest that their interactions are pivotal in shaping both the innate and adaptive immune response and limiting pathogen spread [4, 17–19].

Our studies have focused on the role of bovine NK cells in the immune response to Bacille Calmette Guerin (BCG) vaccination, which induces significant protection from *Mycobacterium bovis* infection [20–22]. Vaccination of calves with BCG is a key strategy for control of bovine tuberculosis and understanding the mechanisms by which BCG stimulates protective immunity may enable further targeted strategies for optimal immune induction. Bovine NK cells secrete IFNγ in response to BCG-infected DC [20, 23], migrate from BCG vaccination sites in afferent lymphatic vessels where they show altered functions [21], and are hypothesized to be central to the induction of protective T cell response. In young cattle and humans, where BCG vaccination is most efficacious against tuberculosis (TB) disease, NK cell production of IFNγ is associated with vaccine-induced immunity [22, 24–26]. Significant roles for NK cells in the early response to mycobacterial infection have also been reported [27].

We hypothesized that BCG vaccination of calves would affect the frequency and localization of NK cells in the draining LN. To measure this, LN were excised 24 or 48 h following administration of BCG to naïve calves, and at the same time points after re-vaccination of previously BCG primed calves. The proportion, localization, aggregation, and clustering of NKp46^+^ NK cells within the BCG vaccine-draining LN were assessed. We also measured expression of CD2^+^ (expressed by LN T cells), and the total number of LN cells for comparative purposes.

## Materials and methods

### Animals and ethical statement

Twelve British Holstein (*Bos taurus*) male calves aged approximately 6 months were placed in four groups (*n* = 3 per group; Table 1) and were vaccinated with 0.5 ml (∼1 × 10^6^ colony forming units confirmed by culture [28]) reconstituted BCG Danish (SSI, Denmark) subcutaneously in the left shoulder region. Two groups of calves were re-vaccinated (boosted) with BCG using the same protocol approximately 6 months later. Age-matched healthy calves were non-vaccinated controls. Calves were euthanized by captive bolt, and exsanguination. All experimental protocols were authorized under the UK Animals (Scientific Procedures) Act, 1986, and performed according to Home Office guidelines with approval from the Roslin Institute animal welfare and ethics review panel. The results are reported in line with the Animal Research: Reporting of In Vivo Experiments (ARRIVE) Guidelines [29].

**Table 1.** Calf groups, vaccination status and LN removal time

Calf group (*n* = 3)	Description
Naïve	Not vaccinated
Group 1.1 (G1.1)	Vaccinated once, lymph nodes removed 24 h post vaccination
Group 1.2 (G1.2)	Vaccinated once, lymph nodes removed 48 h post vaccination
Group 2.1 (G2.1)	Vaccinated twice, lymph nodes removed 24 h post vaccination
Group 2.2 (G2.2)	Vaccinated twice, lymph nodes removed 48 h post vaccination

### LN sample preparation

The left and right prescapular LN (*ln. cervicalis superficialis*) were removed 24 or 48 h post-BCG vaccination, or from naïve animals immediately *post-mortem*. One cubic centimetre pieces of LN were placed in optimal cutting temperature (O.C.T.) embedding medium (ThermoScientific), flash frozen on dry ice, and stored at −70°C. The left LN was BCG draining, and the right LN was used as a control.

### Antibodies

All antibodies used were sourced from Bio-Rad and used at pre-determined optimal dilutions or concentrations. The primary antibodies used were: mouse anti-ovine NKp46 (clone Gr13.1, IgG1, 5 μg/ml); mouse anti-bovine CD2 (clone IL-A42, IgG2a, 1:500), mouse anti-bovine signal regulatory protein alpha [(SIRPα), (clone CC149, IgG2b, 1:500)]. For confocal microscopy, specific binding was detected with goat anti-mouse secondary antibodies coupled to AlexaFlour 488, 568, and 647, respectively, all used at 1:1000.

### Immunofluorescence

Six micrometre sections were cut consecutively from each LN using the cryostat CM1900 (Leica), placed on Superfrost Plus glass slides and left to dry overnight at room temperature. The following day, the sections were fixed in ice-cold Acetone for 10 min, left to dry for 15 min and mounted on Shandon Sequenza slide holders. All washes were carried out in phosphate buffered saline (PBS) containing 0.2% bovine serum albumin, and incubations carried out with 100 μl volumes at room temperature, unless indicated otherwise. Non-specific binding was blocked by incubation with 10% normal goat serum (NGS) diluted in PBS for 30 min. Slides were washed and primary anti-bovine antibodies were applied. After overnight incubation of the primary antibodies at 4°C, and three subsequent washing steps, isotype-specific fluorochrome-labelled secondary antibodies were added for 1 h. Dual-colour staining was performed with fluorochromes selected to minimize spectral overlap. Single-colour staining of each of these molecules was used as a control. Unstained tissue sections were subject to the washing and blocking steps of the procedure but no antibodies were applied. Following washing, sections were counterstained with the nucleic acid stain 4'6-diamidino-2-phenylindole (DAPI) dihydrochloride (Sigma). Finally, all sections were mounted with ProLongGold Antifade mountant medium (ThermoFisher), covered with glass coverslips (Menzel-Glaeser) and left to dry overnight. The sections were stored at 4°C and subsequently imaged with fluorescence microscope Leica DMLB and MicroManager v10 using a 20× PL Fluotar objective (NA 0.5). Digital images of the sections were taken with a CoolSnap (Photometrics) camera at 20× magnification and stored as virtual stack .tif files for visualizing and analyzing with Fiji Image J (NCBI). The microscopy imaging was carried out semi-blind (coded treatment groups) and random images across the tissue were taken. For image assessment and display, colour annotation as chosen from the ImageJ look-up table was as follows: NKp46—green, CD2—white, DAPI—blue.

### Image and data analysis: immunofluorescence

From each LN, between five and seven digital images were assessed initially using Fiji ImageJ for the presence and distribution of fluorescence signals. Grids containing 192 squares (16 × 12) were made in Fiji Image J. All images were split into three channels (blue, green, and white) for analysis. Negative control images (DAPI alone) were used to set basal threshold values for the green and white channels for each group of calves (Supplementary Table S1). The grids and respective threshold values were applied to the images and signals above the threshold were considered positive. For DAPI, all of the visible staining was included as positive.

### Immunohistochemistry

To assess tissue integrity and anatomy of the cryosections, duplicate LN tissue sections were stained with haematoxylin and eosin (H&E) using an automated tissue stainer [Autostainer XL (Leica)]. H&E-stained sections were used to define cortex and medulla areas for further analysis. Parallel sections were then stained for analysis of cell surface expressed molecules to define specific cell populations. Six micrometre sections were cut using a cryostat (Leica CM1900), placed on glass slides (Superfrost Plus, ThermoScientific), left to dry at room temperature for 30 min then fixed with acetone at room temperature for 10 min. Slides were then used immediately for staining or kept at −20°C.

The slides were mounted on Shandon Sequenza slide holders (ThermoScientific) followed by a wash with 1× Tris-buffered saline containing 0.05% Tween20. All subsequent washes were performed with PBS, with incubation volumes of 100 μl at room temperature unless indicated otherwise. Slides were incubated in 1% hydrogen peroxide in methanol for 10 min followed by the addition of 5% NGS for 1 h. The slides were washed three times prior to the addition of primary antibody and incubated at 4°C overnight. Control slides were incubated with PBS containing 5% NGS. The slides were washed three times and HRP conjugate [Invitrogen Biotin XX Tyramide SuperBoost™ Kit (ThermoFisher)] diluted 1:100 was added to each slide and incubated for 30 min. Tyramide working solution was added to the slides and incubated for 10 min. The slides were washed three times, Streptavidin HRP (ThermoFisher; 1:400 in PBS) was added and incubated for 30 min. Following a further three washes, 3,3′-diaminobenzidine solution (Vector Laboratories) was added to the slides and incubated for 15 min. The slides were then removed from the slide holders and counterstained by running in tap water, placed in haematoxylin for 10 s, rinsed with tap water, placed in Scotts Tap Water for 10 s and rinsed again. The slides were dehydrated with the Autostainer XL, mounted with ProLongGold Antifade, coverslips added and dried overnight.

### Image and data analysis: immunohistochemistry

After scanning the slides on the NanoZoomer-XR digital slide scanner (C12000-01, Hamamatsu) and obtaining images of the whole slide, the images were viewed using NDP.view2. To set up a template area for selecting target areas, the magnification was set to 10×. A rectangle was then drawn to select a desired image size, and saved (Supplementary Fig. S1). The image was then zoomed back out, and the saved area was then used to select areas on each image without zooming in to avoid bias. Twelve areas were selected in each image, six from the medulla area and six from the cortex area. After selecting the areas, each area was zoomed in to 10× magnification and a snapshot of the image within the rectangle was saved.

These images were then analyzed using ImageJ. The enhance contrast tool in ImageJ was applied at 0.3% along with the equalize histogram option. In order to set and apply a threshold, the channels of the images had to be split and analysis was carried out on the blue channel, which showed the most contrast between staining and background staining. A threshold was set using the negative control images (secondary antibody only), and a lower and upper threshold was set. The lower threshold was to select any cells present and the upper threshold was set to include all basal nuclei staining. Anything below the lower threshold would mean blank areas with no cells and anything above it would include all the cells in that image. Anything above the upper threshold would mean a positive signal. Each group of calves had a slightly different set of thresholds (Supplementary Table S1). The thresholds were applied to each respective image and the % area occupied by anything below the lower threshold (positive signal) and the % area occupied by cells (below upper threshold) were recorded. The reason an upper threshold was set was because in some images (mainly the medulla areas), there were fewer cells and had some areas with no cells. By measuring the area occupied by all cells and the area occupied by positive signals, it was possible to calculate the % of cells occupied by target signals (positive signals divided by all cells × 100%).

### Statistical analyses

The data were analyzed on R (v4.4.0, © 2024 The R Foundation for Statistical Computing) and RStudio (v2024.04.2 build 764, © 2009–24 Posit Software, PBC). *P* < 0.05 was taken to indicate statistical significance. The packages ggplot2 (v3.5.1), wesanderson (v0.3.7), and gridExtra (v2.3) were used to illustrate the immunofluorescence and immunohistochemistry data.

#### Statistical analyses of immunofluorescence data

Expression of NKp46, CD2, and DAPI by cells in the five different groups of calves (Table 1) was assessed. In the non-vaccinated control group, both left and right LN were termed ‘naive’ (no vaccine). In Groups 2–5, the left LN was BCG-draining and the right (contralateral) was the naïve LN. In each case, data from the left and right LN were compared and contrasted with generalized linear mixed-effect (*GLME*) statistical models using the lme4 (v1.1-35.5) and car (v3.1-3) packages. To take into account both the multiple slides and images per calf, and that left and right LN were taken from each calf, the calf which samples had come from was entered as the random effect. Two fixed-effect variables were considered in each model—the five calf groups, whether left or right LN, and any interactions between them. Where differences were found between groups, either overall or between left or right LN, then Tukey *post hoc* pairwise comparisons were carried out using the multcomp package (v1.4-26).

For the analyses of the percentage of NKp46^+^, CD2^+^, and DAPI^+^ cells, *GLME* with binomial errors (*GLME_b_*) were undertaken. Least square logit-transformed means ($\bar{x}$) and 95% confidence intervals (CIs) from the *GLMEb* were calculated using the package emmeans (v1.10.5) and back-transformed to percentages.

In order to investigate whether the expression of NKp46, CD2, and DAPI was occurring randomly or was aggregated in a subset of cells, statistical analyses of the degree of aggregation were undertaken. For each image, *k*, from the negative binomial distribution was calculated. The *k*-value is an inverse measure of aggregation—a low *k*-value indicates a high aggregation of immunofluorescence between cells and a higher *k*-value (>20) indicates immunofluorescence is occurring randomly between cells [30]. *GLME* with gaussian errors (*GLMEp*) were then undertaken on log_10_-transformed *k* values prior to analysis to normalize the residuals, with the least square $\bar{x}$ ±CIs back-transformed to *k* values.

To examine whether the immunofluorescence of NKp46^+^, CD2^+^, and DAPI^+^ cells was clustered together in the LN or was dispersed throughout the LN, Moran’s *I* was calculated. This is the correlation coefficient that measures overall spatial autocorrelation [31] and reports a value that is between −1 and 1. When the value is close to −1, it means there is high dispersion of data, while 1 indicates clustering of values. Values close to 0 mean there is no correlation of data. The spdep package (v1.3-6) was used to calculate Moran’s *I* within R (version 0.6-13). *GLMEg* were then undertaken to analyze variation in Moran’s *I* between groups and LN and to produce least square $\bar{x}$ ± CIs.

#### Statistical analyses of immunohistochemistry data

NKp46 expression in the five different groups, in the left and right LN, and in the cortex or medulla part of the LN, was compared and contrasted with *GLME_b_* statistical models as above with an additional fixed effect—cortex/medulla location.

## Results

### BCG vaccination increased the frequency of NK cells within the draining LN

**Figure 1.**
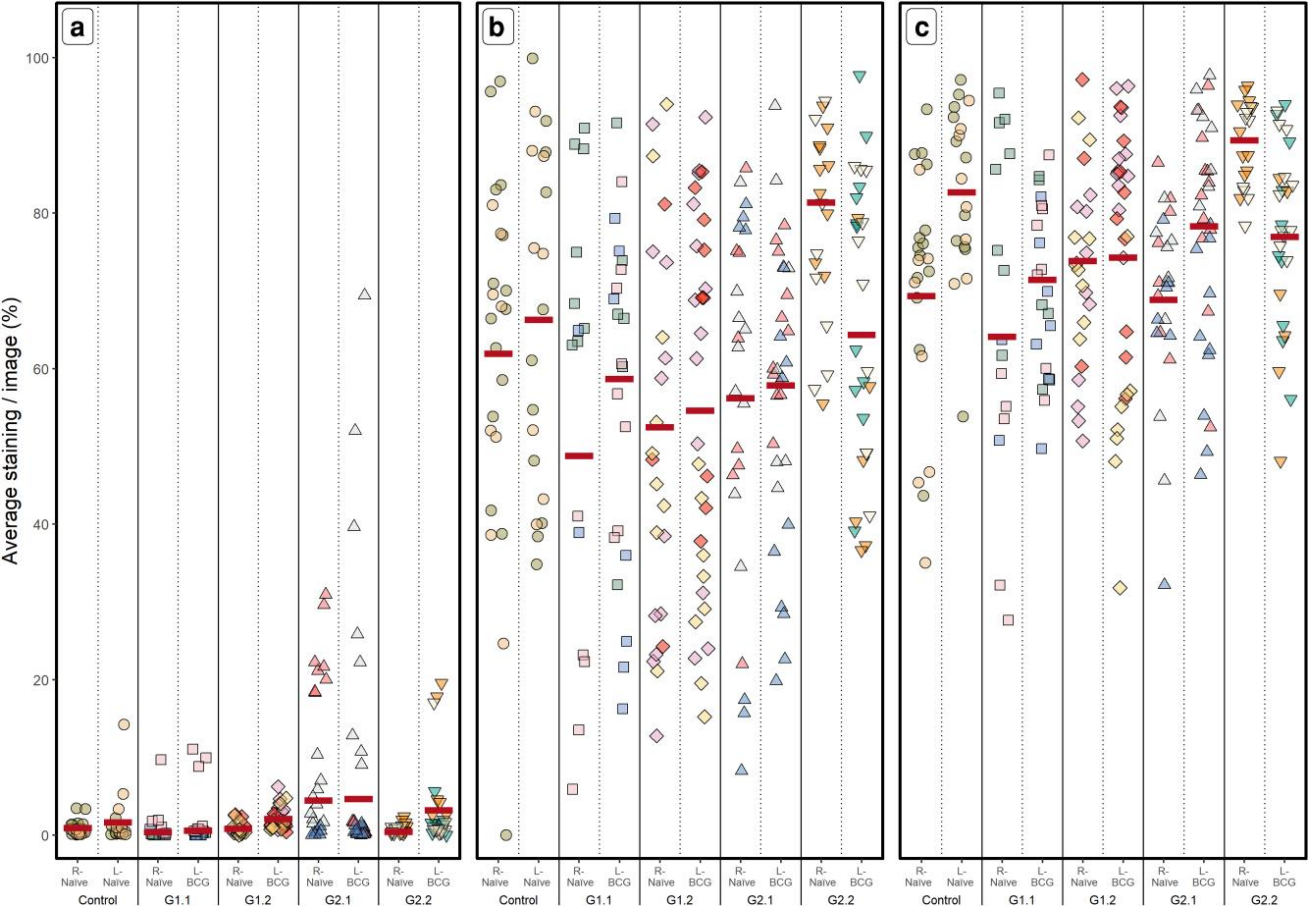
Frequency of cells within bovine LN. Calves were either non-vaccinated (naïve) or vaccinated with BCG into the left shoulder at various time points (Table 1). Five groups of calves are shown; for each group the left (BCG) and right (naive) LN were assessed for expression of NKp46 (a) and CD2 (b) by immunofluorescence, or were stained with DAPI (c). Shown are individual value plots of the average percentage of cells within a sample/image. Individual calves are indicated by different colours and groups by different symbols (circle, naïve; square, G1.1; diamond, G1.2; triangle, G2.1; inverted triangle, G2.2). Horizontal lines indicate the back-transformed least square mean logit estimates from the *GLME_b_* statistical model.

We hypothesized that changes in the frequency, aggregation, and/or clustering of NKp46^+^ cells would occur enabling enhanced interactions with other immune cells within the LN. Using immunofluorescence staining of sequential sections of the LN described above, expression of NKp46 and CD2 were measured (Fig. 1a and b). Uptake of DAPI was used to measure the total cells within the LN (Fig. 1c).

The mean frequency of NKp46^+^ cells across all the samples was 1.62% (CI: 0.8–3.1%, Fig. 1a). There were no differences observed between the five groups overall (*P* = 0.117, $\bar{x}$ = 0.5–4.6%), or between groups when comparing the right (*P* = 0.258, $\bar{x}$ = 0.6–37%) or left LN (*P* = 0.452, $\bar{x}$ = 0.5–3.2%).

We then compared the frequency of NK cells in the LN draining BCG vaccination sites to the frequency observed in the naïve contralateral LN considering all groups of calves together. A higher frequency of NKp46^+^ cells was observed overall in LN post-BCG vaccination (*P* < 0.001), though the overall difference compared with the naïve contralateral LN was relatively small [BCG vaccination $\bar{x}$ = 1.95% (CI: 0.9–4.1), contralateral $\bar{x}$ = 1.31% (CI: 0.6–2.8)].

When analyzing the groups separately, it was evident that BCG had a significant impact on the frequency of NK cells in the vaccine-draining LN compared with the naïve contralateral LN following the primary vaccination in G1.1 and G1.2 (*P* < 0.001), but a lower effect was seen in animals boosted with BCG (G2.1 and G2.2; *P* > 0.071, Table 2).

**Table 2.** Percentage of cells in bovine LN

Group	Lymph node^a^	$\bar{x}$ percentage (95% CI)		
		NKp46	CD2	DAPI
Control^b^	Naïve (R)	0.92 (0.22–3.87)	61.95 (50.43–72.26)	69.36 (60.16–77.24)
	Naïve (L)	1.66 (0.39–6.80)	66.28 (55.12–75.88)	82.66 (76.06–87.74)
G1.1	Naïve (R)^c^	0.41 (0.12–1.38)	48.77 (39.31–58.32)	64.12 (56.19–71.36)
	BCG (L)	0.60 (0.18–1.97)	58.67 (49.15–67.59)	71.44 (64.24–77.70)
G1.2	Naïve (R)	0.84 (0.26–2.74)	52.48 (42.92–61.86)	73.84 (66.95–79.72)
	BCG (L)	2.08 (0.64–6.54)	54.61 (45.04–63.85)	74.31 (67.50–80.10)
G2.1	Naïve (R)	4.50 (1.41–13.42)	56.20 (46.64–65.33)	68.86 (61.36–75.48)
	BCG (L)	4.68 (1.47–13.92)	57.87 (48.33–66.85)	78.32 (72.19–83.42)
G2.2	Naïve (R)	0.48 (0.14–1.57)	81.36 (74.80–86.52)	89.37 (85.75–92.15)
	BCG (L)	3.23 (1.00–9.89)	64.34 (55.13–72.60)	76.94 (70.55–82.29)

^a^Prescapular LN assessed by immunofluorescence for expression of NKp46, CD2 or DAPI staining.

^b^Calves were unvaccinated (control), or vaccinated with BCG and LN removed at 24 (G1.1) or 48 h (G1.2). Groups 2.1 and 2.2 were vaccinated and then re-vaccinated with BCG; LN were removed 24 (G2.1) or 48 h (G2.2) later.

^c^In Groups G1.1, G1.2, G2.1, and G2.2 the left (L) LN drained the BCG vaccination site, the right (R) contralateral LN was considered naïve.

Expression of CD2 was much higher overall when compared with expression of NKp46 [$\bar{x}$ = 60% (CI: 54.7–65.1), Fig. 1b], in line with the expected expression of CD2 on T cells but not on NK cells in the LN. However, the impact of BCG vaccination on the frequency of CD2 expression varied between groups (Fig. 1b). Overall, BCG did not significantly affect the frequency of CD2 expression, although the relationship between calf group and LN was variable (Table 2). Similarly, high-level DAPI expression was observed [$\bar{x}$ = 75% (CI: 71.2–78.5), Fig. 1c], with variability between groups and no consistent association with BCG vaccination status (Fig. 1c; Table 2).

### Differences in the aggregation and clustering of NK cells were not observed following BCG vaccination

**Figure 2.**
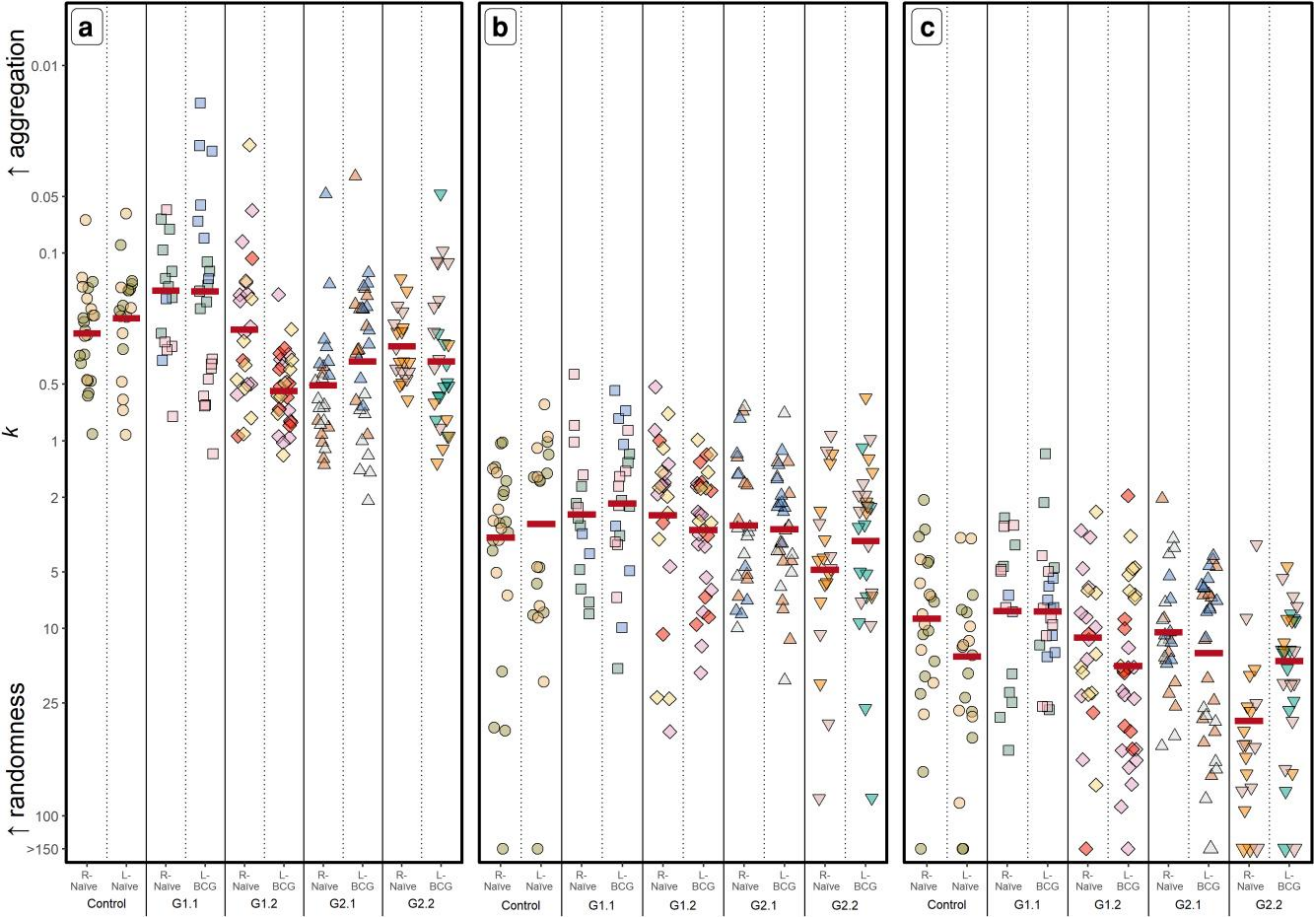
Aggregation of cells within bovine LN. Calves were vaccinated and LN removed as described for Fig. 1. Expression of NKp46 (a) and CD2 (b) were examined by immunofluorescence. Cell nuclei were stained with DAPI (c). The degree of aggregation (*k*) of expression of (a) NKp46; (b) CD2; (c) DAPI is shown by group and LN type as described for Fig. 1. Horizontal lines indicate the back-transformed least square mean log_10_-transformed *k* estimates from the *GLME_g_* statistical model.

Overall, there was greater aggregation of NKp46 expression in a subset of cells within the LN [$\bar{x}$*k* = 0.30 (CI: 0.21–0.43), Fig. 2a] compared with CD2 [$\bar{x}$*k* = 3.30 (CI: 2.49–4.37), Fig. 2b] indicating that, even in non-vaccinated animals, NKp46 expression occurs in a subset of cells in the prescapular LN. In contrast, DAPI staining was randomly distributed [$\bar{x}$*k* = 13.9 (CI: 10.7–18.1), Fig. 2c]. No overall significant differences were observed in the aggregation of NKp46 expression in cells between the five groups ($\bar{x}$*k* = 0.16–0.43, *P* = 0.134) or whether the left or right LN was being considered ($\bar{x}$*k* = 0.29 and 0.32, *P* = 0.294). In addition, within G1.1, G2.1, and G2.2, no differences in the aggregation of NKp46 expression were observed irrespective of whether calves were vaccinated (or boosted) with BCG (*P* > 0.102, Table 3). The only difference observed was relatively less aggregation of NKp46 expression in the BCG-draining LN in G1.2 [$\bar{x}$*k* = 0.54 (CI: 0.39–0.74)], compared with the naïve, contralateral LN [$\bar{x}$*k* = 0.25 (CI: 0.18–0.36), *P* < 0.001]. No consistent differences in the degree of aggregation of CD2 or DAPI expression between LN were observed overall or within groups (*P* > 0.097, Table 3).

**Table 3.** Aggregation and clustering of cells in bovine LN represented as *k* and Moran’s *I*

Group	Lymph node^a^	$\bar{x}$ *k* (95% CI)			$\bar{x}$ Moran’s *I* (95% CI)		
		NKp46	CD2	DAPI	NKp46	CD2	DAPI
Control^b^	Naïve (R)	0.27 (0.12–0.62)	3.28 (2.12–5.10)	8.86 (5.45–14.42)	0.24 (0.05–0.44)	0.64 (0.56–0.72)	0.57 (0.51–0.63)
	Naïve (L)	0.22 (0.10–0.52)	2.77 (1.74–4.40)	14.16 (8.53–23.51)	0.26 (0.06–0.45)	0.65 (0.57–0.73)	0.61 (0.55–0.68)
G1.1	Naïve (R)^c^	0.16 (0.08–0.33)	2.47 (1.50–4.05)	8.11 (4.90–13.41)	0.17 (0.01–0.33)	0.56 (0.48–0.65)	0.49 (0.42–0.56)
	BCG (L)	0.16 (0.08–0.32)	2.16 (1.42–3.29)	8.13 (5.25–12.60)	0.27 (0.10–0.43)	0.71 (0.64–0.78)	0.57 (0.51–0.63)
G1.2	Naïve (R)	0.26 (0.13–0.52)	2.50 (1.64–3.78)	11.22 (7.16–17.60)	0.28 (0.12–0.44)	0.61 (0.54–0.68)	0.58 (0.53–0.64)
	BCG (L)	0.54 (0.27–1.08)	2.99 (2.07–4.32)	15.84 (10.56–23.76)	0.32 (0.16–0.48)	0.60 (0.53–0.66)	0.51 (0.46–0.56)
G2.1	Naïve (R)	0.51 (0.25–1.02)	2.83 (1.90–4.20)	10.51 (6.90–16.02)	0.45 (0.29–0.61)	0.64 (0.57–0.72)	0.53 (0.47–0.58)
	BCG (L)	0.38 (0.19–0.75)	2.96 (2.05–4.26)	13.55 (9.05–20.30)	0.38 (0.22–0.54)	0.63 (0.56–0.70)	0.59 (0.54–0.64)
G2.2	Naïve (R)	0.31 (0.15–0.65)	4.88 (3.06–7.78)	31.09 (18.65–51.85)	0.20 (0.03–0.36)	0.61 (0.53–0.69)	0.54 (0.48–0.61)
	BCG (L)	0.38 (0.19–0.76)	3.42 (2.34–5.02)	14.93 (9.82–22.71)	0.34 (0.18–0.50)	0.63 (0.56–0.70)	0.53 (0.48–0.59)

^a^Prescapular LN assessed by immunofluorescence for expression of NKp46, CD2 or DAPI staining.

^b^Calves were unvaccinated (control), or vaccinated with BCG and LN removed at 24 (G1.1) or 48 h (G1.2). Groups 2.1 and 2.2 were vaccinated and then re-vaccinated with BCG; LN were removed 24 (G2.1) or 48 h (G2.2) later.

^c^In Groups G1.1, G1.2, G2.1, and G2.2 the left (L) LN drained the BCG vaccination site, the right (R) contralateral LN was considered naïve.

**Figure 3.**
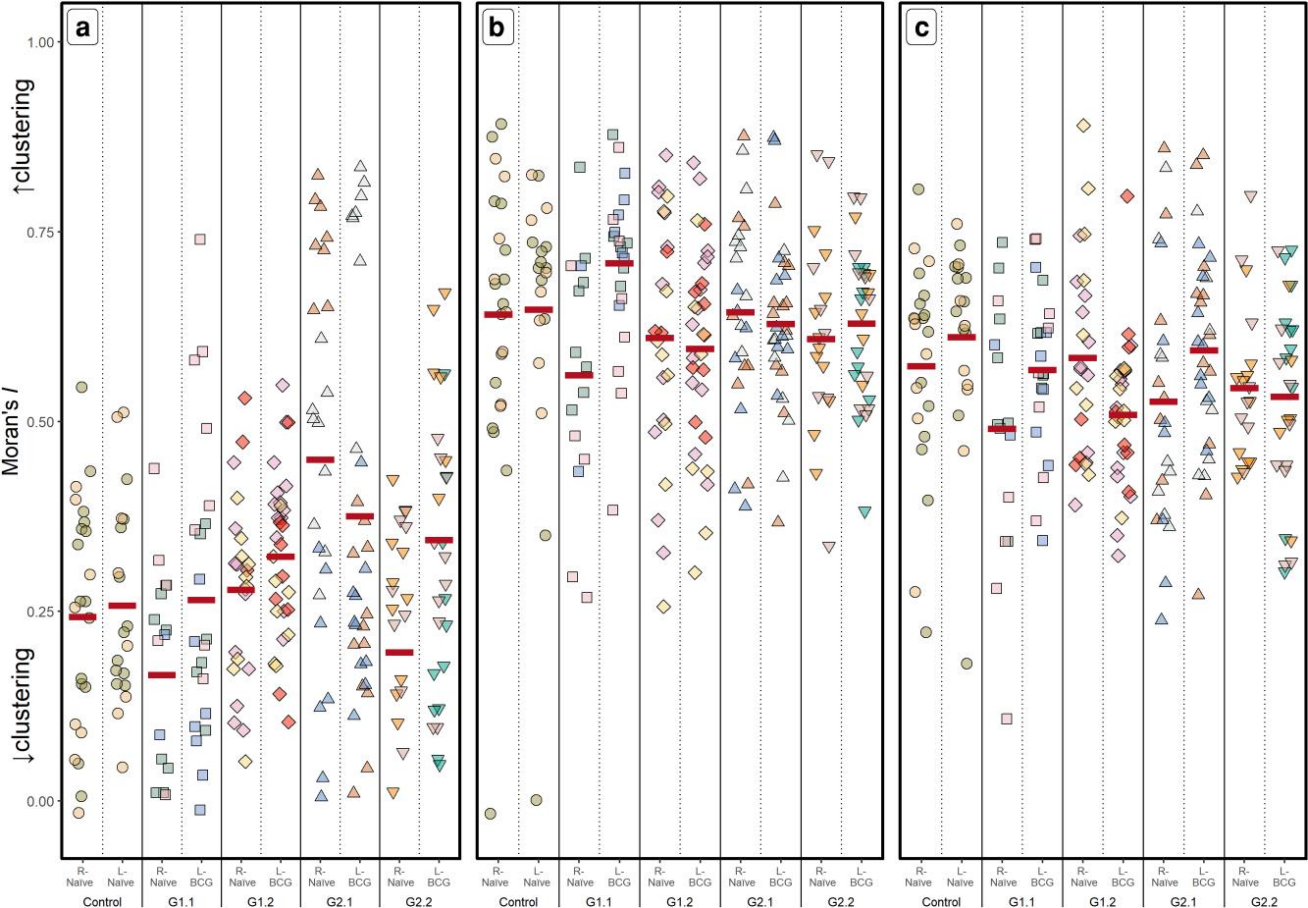
Clustering of cells within bovine LN. Calves were vaccinated and LN removed as described for Fig. 1. Expression of NKp46 (a), CD2 (b) were examined by immunofluorescence and cell nuclei were stained with DAPI (c). The degree of clustering (Moran’s *I*) within the LN of expression of (a) NKp46; (b) CD2; (c) DAPI is shown by group and LN type as described for Fig. 1. Horizontal lines indicate the least square mean Moran’s *I* estimates from the *GLME_g_* statistical model.

Assessment of Moran’s *I* values (Fig. 3) revealed that in naïve LN, NKp46^+^ cells [$\bar{x}$  *I* = 0.25 (CI: 0.07–0.43), Fig. 3a] were less clustered within the LN than CD2^+^ cells [$\bar{x}$  *I* = 0.64 (CI: 0.57–0.71), Fig. 3b] or the total cell population [$\bar{x}$  *I* = 0.59 (CI: 0.54–0.65), Fig. 3c]. There was high variability in the Moran’s *I* values for all three cell populations between samples from the same calves. Overall, no significant differences were observed between non-vaccinated and BCG-vaccinated animals ($\bar{x}$ NKp46^+^  *I* = 0.23–0.41; $\bar{x}$ CD2^+^  *I* = 0.60–0.65, $\bar{x}$ DAPI^+^  *I* = 0.54–0.56; *P* > 0.276), or between BCG-draining LN and the naïve contralateral LN ($\bar{x}$ NKp46 *I* = 0.29–0.32; $\bar{x}$ CD2 *I* = 0.61–0.64, $\bar{x}$ DAPI *I* = 0.54–0.55; *P* > 0.122). Within groups, there was little difference in the clustering between LN (Table 3).

### The anatomical distribution of NKp46^+^ NK cells within the prescapular LN was not altered following BCG vaccination

**Figure 4.**
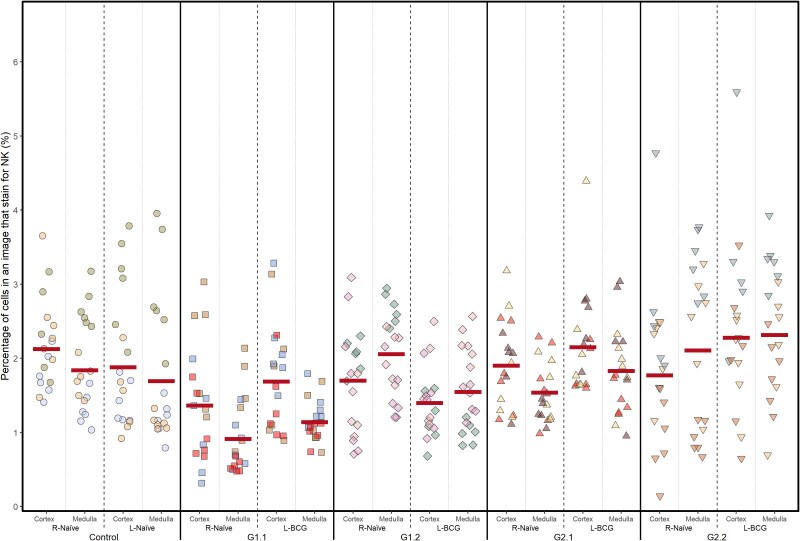
Frequency of NKp46^+^ cells within cortex and medulla of bovine LN. Calves were vaccinated and LN removed as described for Fig. 1. Expression of NKp46 in the cortex and medulla of the LN were examined by immunohistochemistry. Shown are individual value plots of the average percentage of cells within a sample/image taken from LN that express NKp46, divided by group (naïve, G1.1-G2.2), whether the LN was the BCG-draining or the contralateral (naive) LN, and where in the LN images were captured (cortex or medulla). Individual calves are indicated by different colours and samples from calves in the different groups by filled symbols (circle, naïve; square, G1.1; diamond, G1.2; triangle, G2.1; inverted triangle, G2.2). Horizontal lines indicate the back-transformed least square mean logit estimates from the *GLME_b_* statistical model.

The presence of NKp46^+^ cells within prescapular LN was further assessed by immunohistochemistry. The left and right LN were examined for each animal, and the frequency (%) of cells was measured in the medulla and cortex areas. In non-vaccinated animals, low frequencies of NKp46^+^ cells [$\bar{x}$ = 1.7% (CI: 1.5–2.0)] were observed, with no significant differences between the cortex and medulla [$\bar{x}$ = 2.0% (CI: 1.5–2.8) vs. $\bar{x}$ = 1.8% (CI: 1.34–2.4), *P* = 0.057], nor between the left and right LN [$\bar{x}$ = 1.8% (CI: 1.3–2.5) vs. $\bar{x}$ = 2.0% (CI: 1.4–2.7), *P* = 0.123, Fig. 4]. However, for BCG-vaccinated animals, some differences in NK cell frequency between the cortex and medulla, as well as between the BCG-vaccinated LN and the naïve contralateral LN were observed. These differences varied with group (*P* < 0.001). No consistent alterations were detected in the frequency of NK cells following BCG vaccination when considering their location in the cortex or medulla of the LN (Table 4, Fig. 4).

**Table 4.** Percentage of NKp46^+^ NK cells in bovine LN by group, and anatomical region

Group	Lymph node^a^	Location	$\bar{x}$ percentage (95% CI)
			NKp46
Control^b^	Naïve (R)	Cortex	2.12 (1.63–2.77)
		Medulla	1.84 (1.40–2.40)
	Naïve (L)	Cortex	1.88 (1.43–2.46)
		Medulla	1.69 (1.29–2.22)
G1.1	Naïve (R)^c^	Cortex	1.36 (1.03–1.80)
		Medulla	0.91 (0.68–1.23)
	BCG (L)	Cortex	1.69 (1.28–2.21)
		Medulla	1.14 (0.86–1.51)
G1.2	Naïve (R)	Cortex	1.70 (1.29–2.24)
		Medulla	2.06 (1.57–2.69)
	BCG (L)	Cortex	1.40 (1.05–1.85)
		Medulla	1.55 (1.17–2.04)
G2.1	Naïve (R)	Cortex	1.90 (1.45–2.49)
		Medulla	1.54 (1.17–2.02)
	BCG (L)	Cortex	2.15 (1.65–2.80)
		Medulla	1.83 (1.40–2.39)
G2.2	Naïve (R)	Cortex	1.77 (1.35–2.31)
		Medulla	2.10 (1.62–2.73)
	BCG (L)	Cortex	2.28 (1.76–2.95)
		Medulla	2.31 (1.78–3.00)

^a^Prescapular LN assessed by immunofluorescence for expression of NKp46, CD2, or DAPI staining.

^b^Calves were unvaccinated (control), or vaccinated with BCG and LN removed at 24 (G1.1) or 48 h (G1.2). Groups 2.1 and 2.2 were vaccinated and then re-vaccinated with BCG; LN were removed 24 (G2.1) or 48 h (G2.2) later.

^c^In Groups G1.1, G1.2, G2.1, and G2.2 the left (L) LN drained the BCG vaccination site, the right (R) contralateral LN was considered naïve.

## Discussion

There is a growing body of evidence for the importance of NK cells in protective immunity, and resistance to TB. In humans, cattle, non-human primates, and mice, studies of the mechanisms by which BCG provides protective immunity point to significant roles for NK cells [22, 24, 32–34]. NK cells secrete key cytokines such as IFNγ, and may also exert direct cytotoxic effects on infected cells that contribute to control of mycobacteria. They also contribute to trained immunity [35, 36], and memory-like responses that are characterized by enhanced IFNγ responses upon re-stimulation or secondary antigen exposure [37–39]. The capacity of NK cells to secrete IFNγ, and their reciprocal interaction with DCs, has been shown to significantly influence T cell activation: this is crucial for protective immunity against a range of pathogens including mycobacteria [6, 14, 40, 41]. In line with this, vaccine adjuvants have been shown to significantly enhance NK recruitment to draining LN. This was associated with both increased IFNγ expression by NK cells and IL-12 secretion by DCs, that are required for Th1-biased immunity [6, 15]. The influence of NK cells on CD8^+^ T cell activation by adjuvants has also been reported, and an impact on cDC1 spatio-temporal distribution through NK cell interactions also likely influence CD8^+^ T cell activation [42]. Taken together, these studies highlight a central role for NK cells in vaccine immunity. We previously showed that BCG vaccination altered the frequency and phenotype of NK cells in afferent lymphatics draining BCG vaccination sites [21], and that the majority of NK cells in lymph were of the NKp46^+^CD2^−^ phenotype with high-level IFNγ-secreting capacity [43, 44]. Moreover, the bovine NKp46^+^CD2^−^ NK cells interacted uniquely with BCG and *M. bovis*-infected DCs producing IFNγ under the influence of DC-derived IL-12 in reciprocal interactions that could enhance T cell activation [20, 23]. We hypothesized here that BCG vaccination of cattle would alter the frequency and distribution of NK cells leading to spatial alterations that would enhance cellular cross-talk with DCs and T cells for protective immune response induction. Understanding the mechanisms by which BCG impacts NK cells could aid identification of adjuvants to improve the induction of protective immunity following vaccination.

Studies of the impact of vaccination on bovine LN NK cells may be hampered by the pre-existing high frequency of these cells within naïve LN [12], that are already distributed in areas where both DCs and T cells reside [12]. In naïve (laboratory) mice, naïve LN are virtually free of NK cells [4–6], and identifying small changes in frequency may therefore be more straightforward. Nevertheless, we were able here to demonstrate significant alterations in NK cell frequency following BCG vaccination, particularly on the first (priming) vaccination with BCG. Although the effect was lower in LN following a re-vaccination with BCG, this was not unexpected as second exposure may be more likely to influence the function of NK cells, particularly IFNγ secretion [45], rather than further recruitment to the LN. In mice, Saxena *et al*. [46] demonstrated that intranasal BCG vaccination enhanced the number of NK cells within draining LN, and the administration of mycobacterial antigens was also shown to increase percentages of activated NK cells within localized lymphoid tissues [16].

In our study, although BCG increased the frequency of NK cells within draining LN, there was no evidence for redistribution of these cells within the node. This may reflect that, even in the steady state, NK cells are located in proximity to DCs and T cells in bovine LN with scattered distribution throughout the LN. In mice, redistribution of NK cells to more central regions of the parafollicular areas likely influences T cell activation and interactions with DC. The NK cells in these activated LN were significantly more mobile resulting in wide-spread distribution and enabling multiple transient interactions with DCs [8, 16]. Furthermore, depletion of NK cells altered the frequency of DCs, macrophages, and neutrophils present in draining LN early following administration of mycobacterial antigens and this was associated with lower expression of IL-22, tumour necrosis factor alpha (TNFα) and IFNγ by antigen-specific T cells in the periphery [16]. Recent evidence for specific roles of CD8α^+^ NK cells in TB immunity [47] and resistance [48] suggest that investigating specific subsets of NK cells may reveal further insights. We focused here on NKp46^+^ cells, but NKp46-negative NK cells may also be of functional importance [49]. Further studies are required in cattle to examine the spatial distribution of NK cell subsets alongside DCs and T cells, and to determine functional alterations that drive immunity. Utilizing spatial transcriptomics and proteomics, alongside multicolour immunocytochemistry will be essential to define the mechanisms by which NK cells may directly, or indirectly influence BCG-induced immunity through their interactions with DCs and T cells. This would enable proximity assessments of these cells, and analysis of molecular pathways that could influence their interactions within the tissues. Combined with *in vitro* analyses of cellular cross-talk [23, 41], this would facilitate greater understanding of the role of NK cells in immunity.

## Supplementary Material

kyaf017_Supplementary_Data

## Data Availability

The data underlying this article will be shared on reasonable request to the corresponding author.
