# Supplementary material for: Vaccination of calves with Bacille Calmette Guerin increased the frequency but did not affect aggregation or clustering of natural killer cells in draining lymph nodes
Source: Discov Immunol. 2025 Nov 13;4(1):kyaf017. doi: 10.1093/discim/kyaf017 (PMC12772509; doi:10.1093/discim/kyaf017)
Supplement: kyaf017_Supplementary_Data [file kyaf017_supplementary_data.zip › Supplementary Table.docx]

(a)

| **Group** | **Green** | **White** |
| --- | --- | --- |
| **Naïve** | 1853 | 272 |
| **1.1** | 1962 | 264 |
| **1.2** | 1471 | 254 |
| **2.1** | 1536 | 244 |
| **2.2** | 1471 | 241 |

(b)

|  | **Naive** | **G1.1** | **G1.2** | **G2.1** | **G2.2** |
| --- | --- | --- | --- | --- | --- |
| **Lower** | 37 | 33 | 32 | 33 | 33 |
| **Upper** | 204 | 215 | 206 | 213 | 218 |

*Supplementary Table 1: Threshold values used for immunofluorescent staining (a) and IHC (b) analysis from Image J.*
