# Supplementary figures and images for: Vaccination of calves with Bacille Calmette Guerin increased the frequency but did not affect aggregation or clustering of natural killer cells in draining lymph nodes
Source: Discov Immunol. 2025 Nov 13;4(1):kyaf017. doi: 10.1093/discim/kyaf017 (PMC12772509; doi:10.1093/discim/kyaf017)

## Slide 1
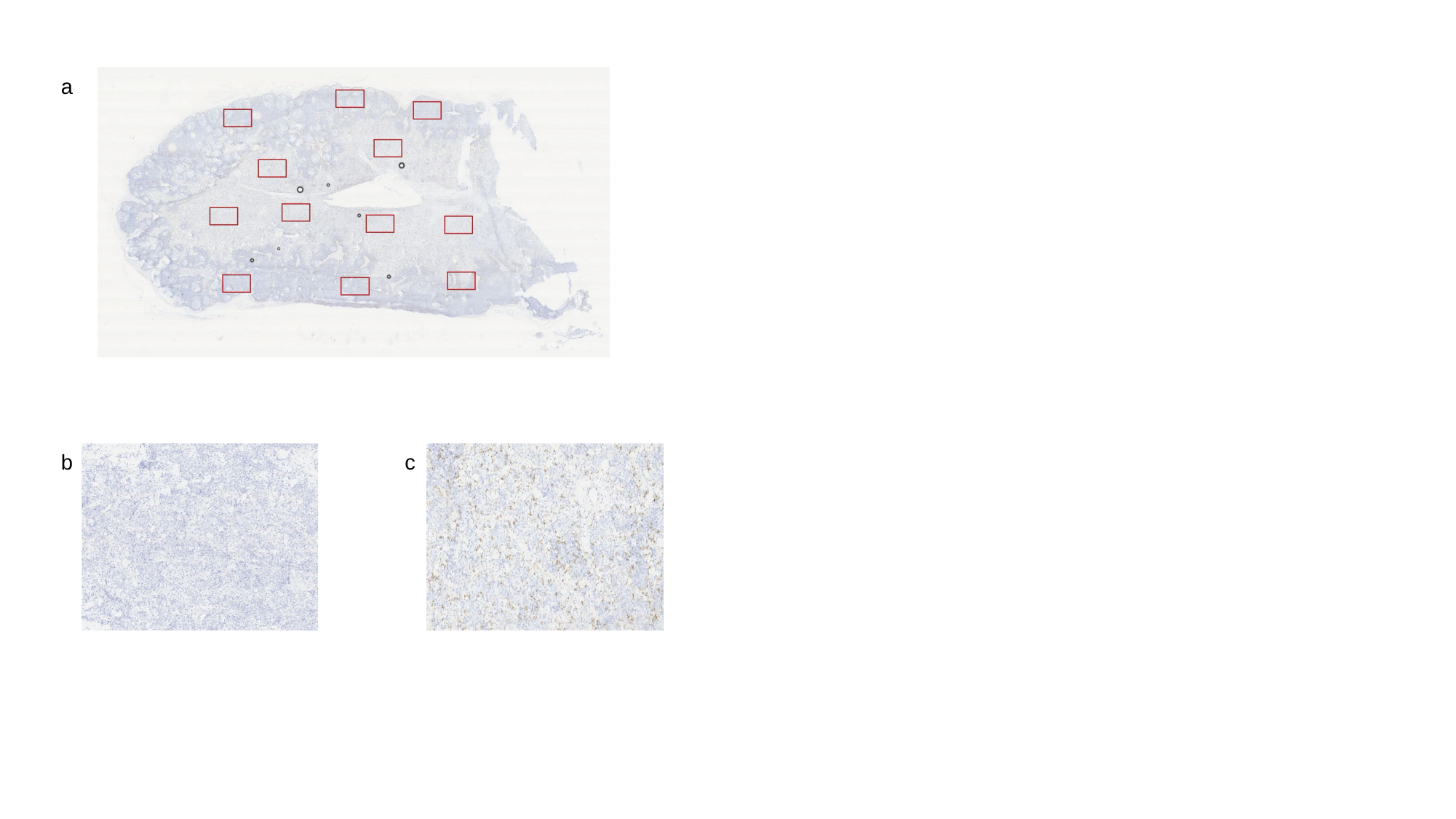

a
c
b

Supplement: kyaf017_Supplementary_Data [file kyaf017_supplementary_data.zip › Supplementary Figure 1_Hope 2025.pptx]
